# HIV self-test performance evaluation among priority populations in rural Mozambique: Results from a community-based observational study

**DOI:** 10.1371/journal.pone.0305391

**Published:** 2024-06-17

**Authors:** Caroline De Schacht, Carlota Lucas, Paula Paulo, Anibal Naftal Fernando, Jalilo Ernesto Chinai, Wilson P. Silva, Guita Amane, Thebora Sultane, Nely Honwana, Inacio Malimane, Aleny Couto, Zhihong Yu, C. William Wester

**Affiliations:** 1 Friends in Global Health (FGH), Maputo, Mozambique; 2 Friends in Global Health (FGH), Quelimane, Mozambique; 3 Provincial Health Directorate of Zambézia (DPS-Z), Quelimane, Mozambique; 4 National Directorate for Public Health, Ministry of Health (MOH), Maputo, Mozambique; 5 Instituto Nacional de Saúde (INS), Marracuene, Mozambique; 6 Division of Global HIV & TB, U.S. Centers for Disease Control and Prevention (CDC), Maputo, Mozambique; 7 Department of Biostatistics, Vanderbilt University Medical Center (VUMC), Nashville, Tennessee, United States of America; 8 Vanderbilt Institute for Global Health (VIGH), Nashville, Tennessee, United States of America; 9 Department of Medicine, Division of Infectious Diseases, Vanderbilt University Medical Center (VUMC), Nashville, Tennessee, United States of America; Eduardo Mondlane University: Universidade Eduardo Mondlane, MOZAMBIQUE

## Abstract

**Background:**

In 2021, Mozambique initiated community-based oral HIV self-testing (HIVST) to increase testing access and uptake among priority groups, including adult males, adolescents, and young adults. Within an HIVST pilot project, we conducted a performance evaluation assessing participants’ ability to successfully conduct HIVST procedures and interpret results.

**Methods:**

A cross-sectional study was performed between February-March 2021 among employees, students (18–24 years of age), and community members, using convenience sampling, in two rural districts of Zambézia Province, Mozambique. We quantified how well untrained users performed procedures for the oral HIVST (Oraquick^®^) through direct observation using a structured checklist, from which we calculated an HIVST usability index (scores ranging 0–100%). Additionally, participants interpreted three previously processed anonymous HIVST results. False reactive and false non-reactive interpretation results were presented as proportions. Bivariate analysis was conducted using Chi-square and Fisher exact tests.

**Results:**

A total of 312 persons participated (131[42%] community members, 71[23%] students, 110[35%] employees); 239 (77%) were male; the mean age was 28 years (standard deviation 10). Average usability index scores were 80% among employees, 86% among students, and 77% among community members. Main procedural errors observed included “incorrect tube positioning” (49%), “incorrect specimen collection” (43%), and “improper waiting time for result interpretation” (42%). From the presented anonymous HIVST results, 75% (n = 234) correctly interpreted all three results, while 9 (3%) of study participants failed to correctly interpret any results. Overall, 36 (12%) gave a false non-reactive result interpretation, 21 (7%) a false reactive result interpretation, and 14 (4%) gave both false non-reactive and false reactive result interpretations. Community members generally had lower performance.

**Conclusions:**

Despite some observed testing procedural errors, most users could successfully perform an HIVST. Educational sessions at strategic places (*e*.*g*., schools, workplaces), and support via social media and hotlines, may improve HIVST performance quality, reducing the risk of incorrect interpretation.

## Introduction

HIV self-testing (HIVST) is a strategy recommended by the World Health Organization (WHO) to enhance HIV testing uptake rates [1, 2], especially among groups that are traditionally more difficult to reach. HIVST can be performed assisted or unassisted, however, it is necessary to seek services to confirm a reactive HIVST result, as a single rapid test (*i*.*e*., HIVST) is not sufficient to make an HIV positive diagnosis. Studies evaluating HIVST performance among specific priority populations such as female sex workers (FSW), as well as the general population, including males and young adults are usually country-specific [3–8]. Variability in HIVST performance has been observed based on the HIVST sample type, namely, whole blood versus oral saliva. In the past, slightly poorer test performance was seen with oral saliva-based samples, with a lower sensitivity and lower positive predictive value (PPV) when evaluated in low prevalence settings [9]. However, subsequent studies performed in high HIV prevalence settings have now shown comparable test performance with oral saliva and whole blood based samples, with excellent PPV’s being reported [10, 11]. It also is important to continuously monitor HIVST performance in order to tailor information, educational needs, and communication strategies at the community level.

Mozambique, a geographically large country with an estimated 65% of the population living in rural areas, has an HIV prevalence of 12.5%, according to the most recent national HIV impact assessment in 2021 [12]. The 2015 national HIV survey showed that 39% of women and 60% of men had never been tested for HIV [13]. To increase HIV testing coverage, the country initiated an HIVST pilot project in one province in the central region of the country in 2019 (Zambézia Province); results have been published elsewhere [14]. Following the pilot, the Ministry of Health (MOH) elaborated national HIVST guidelines and began expanding its implementation in 2021 [15]. The MOH further piloted community distribution of HIVST test kits in 2021 and expanded the availability of this testing approach for the general population nationally beginning in 2022, in order to reach those who are generally difficult to reach. The national guidelines recommend confirmation of a reactive HIVST at the health facility. No data exist yet on the quality of HIVST performance in the country. The aim of this study was to evaluate the quality of HIVST performance and result interpretation among employees (who are primarily male in our context), young adults (students), and community members in general, who were each identified as priority groups for HIVST by the MOH.

## Materials and methods

### Study setting

The study was implemented in Zambézia Province, which is situated in the central region of Mozambique and whose large population (~6 million) is mainly rural (82%) [16]. In 2021, based on the recently completed Population-Based HIV Impact Assessment, the adult (≥ 15 years of age) HIV prevalence in Zambézia was 17.1% (95% CI 13.0%-21.2%) [12]. Alto Molócuè and Mocuba, two rural districts in Zambézia, where the HIVST pilot study was implemented, were selected for inclusion in this evaluation. The study populations were recruited from secondary/ superior level schools, small companies in the two districts, and from communities surrounding the two main district health facilities. All schools and companies in the two districts were listed and approached, and those interested in the project were included in the study, resulting in inclusion of 3 schools and 5 companies.

### Study design and study population

We used a cross-sectional study design and recruited participants from the following groups: employees, students and community members. Inclusion criteria were: 1) for all participants: willingness to know their HIV status and a minimum age of 18 years; 2) for employees: adult, salaried employee for at least one month at a registered company in the select districts; 3) for students: 18 to 24 years of age and studying in one of the recognized tertiary educational institutions in the selected districts; 4) for community members: individuals living in the selected districts (who do not otherwise qualify as an “employee” or “student”). Individuals with a self-reported known HIV-positive status were excluded. The groups were selected to capture different populations: males (employees), young adults (students), and the general population (community members).

### Data collection

Data were collected between February and March 2021. The research staff convened group information sessions for managers of the selected companies and directors of selected schools and institutions prior to the data collection activities to provide information about the study objectives and procedures; the managers and directors then informed their employees and students respectively regarding the study. For implementation in the community, community leaders were invited to participate in information sessions to learn more about the objectives and procedures of the study. Subsequently, the leaders disseminated information about the study within the community. Recruitment was done through convenience sampling, as per availability and interest on the date of data collection. Interested individuals were referred to designated places within respective recruitment locations, for example, a meeting room at the company locations, a classroom or meeting room at the schools, or a meeting room in a nearby health facility or school for community members. The research assistants explained about the study and completed the informed consent process with the interested individuals. Consenting participants were evaluated on their performance of HIVST procedures through observation by independently trained research assistants. The oral HIVST OraQuick HIV1/2^®^ Self-Test (OraSure Technologies Inc., Bethlehem, PA, U.S.) was used through a donation from OraSure Technologies. This test is approved by the U.S. Food and Drug Administration as an oral home HIV test [17] and has been pre-qualified by the WHO [18]. The test entails using an oral swab and no blood samples are needed. The manufacturers’ written instructions were available to participants in Portuguese and a demonstration video of the procedures was offered in Portuguese and in the two main local languages, Chuabo, and Manhaua, for those interested. The video was adapted from the manufacturers’ video *(*http://www.oraquick.com/Taking-the-Test/How-To-Video*)* to local context and language. Six trained research assistants watched how participants performed the HIV self-test, without interfering or responding to any question related to the testing procedures (one assistant per participant). The research assistants used a structured checklist with a total of 20 checklist items (13 procedure-related items, 7 supportive items) based on other performance evaluations and adapted for the Mozambican context for this study [3, 6]. The research assistant observed and documented the participant’s performance of the testing procedures per the manufacturer’s instructions on the checklist, including the completion (or not) of the four specific testing components: Preparation, Collection, Mixing, and Results Reading. In addition, participants were asked to interpret the results of three previously processed anonymous self-tests. The tests, with either an HIV-non-reactive, HIV-reactive, or invalid results, were shown at random to the participants via photos, to assess the participants’ interpretation of test results. Participants were not required to share their own test result after completing the HIVST and results were not seen by the research assistants.

### Statistical considerations

Performance evaluation sample size was calculated based on pilot studies [8, 19], in which performance without errors was observed 85% of the time. We anticipated that approximately 70% of employees, 85% of students, and 50% of community members would be able to perform the HIVST without errors. Assuming a margin of error (MOE) is equal to half of the Wilson Score interval at 90% confidence, and solving the following equation for n, we estimated that a sample size of at least 55 employees, 35 students, and 65 community members from each district would be sufficient to estimate the probability of success within each district with an MOE of 10%.

$$MOE\;=\frac{z}{1+\frac{z^{2}}{n}}\sqrt{\frac{p\;\times \;\left(1-p\right)}{n}+\frac{z^{2}}{4n^{2}}}$$

Where z is the z-score corresponding to the desired confidence interval, p is the expected proportion of participants who would perform the HIVST without errors, and MOE is the desired margin of error. HIVST usability was calculated as the sum of the scores of each of the 13 observations, where the observations with expected negative (“No” as response) and observations with expected positive (“Yes” as response) responses were counted. The usability percentage for each observation was calculated and an average was provided for the general usability index (UI), ranging between 0% (not useable) to 100% (highly usable). False reactive and false non-reactive interpretations were calculated as follows: a) false reactive: when participants provided an interpretation of “reactive” to a test result that was actually non-reactive or invalid, and b) false non-reactive: when participants provided an interpretation of “non-reactive” to a test result that was actually reactive or invalid. A correct interpretation was defined as follows: providing a “reactive” interpretation when the test result was reactive, providing a “non-reactive” interpretation when the test result was non-reactive, and providing an “invalid” interpretation when the test result was invalid. The Chi-square (or Fisher exact) tests were used for comparison analyses among the three participant groups. Descriptive and comparative analyses were performed in R version 4.0.1 [20].

### Ethical considerations

The protocol and instruments were approved by the institutional health ethics committee of the “Instituto Nacional de Saúde” (INS) (CIBS-INS, reference 080/CIBS-INS/2018), the Vanderbilt University Medical Center (VUMC) Institutional Review Board (IRB) (#181834), and the Mozambique National Directorate of Pharmacies, and was reviewed in accordance with the U.S. Centers for Disease Control and Prevention (CDC) human research protection procedures and was determined to be research. The CDC co-investigators did not interact with human subjects or have access to identifiable data or specimens for research purposes. Informed consent was administered by research assistants speaking the local language(s). All participants provided written informed consent prior to study participation. Additional information regarding the ethical, cultural, and scientific considerations specific to inclusivity in global research is included in the Supporting information (S1 File).

## Results

**Fig 1:**
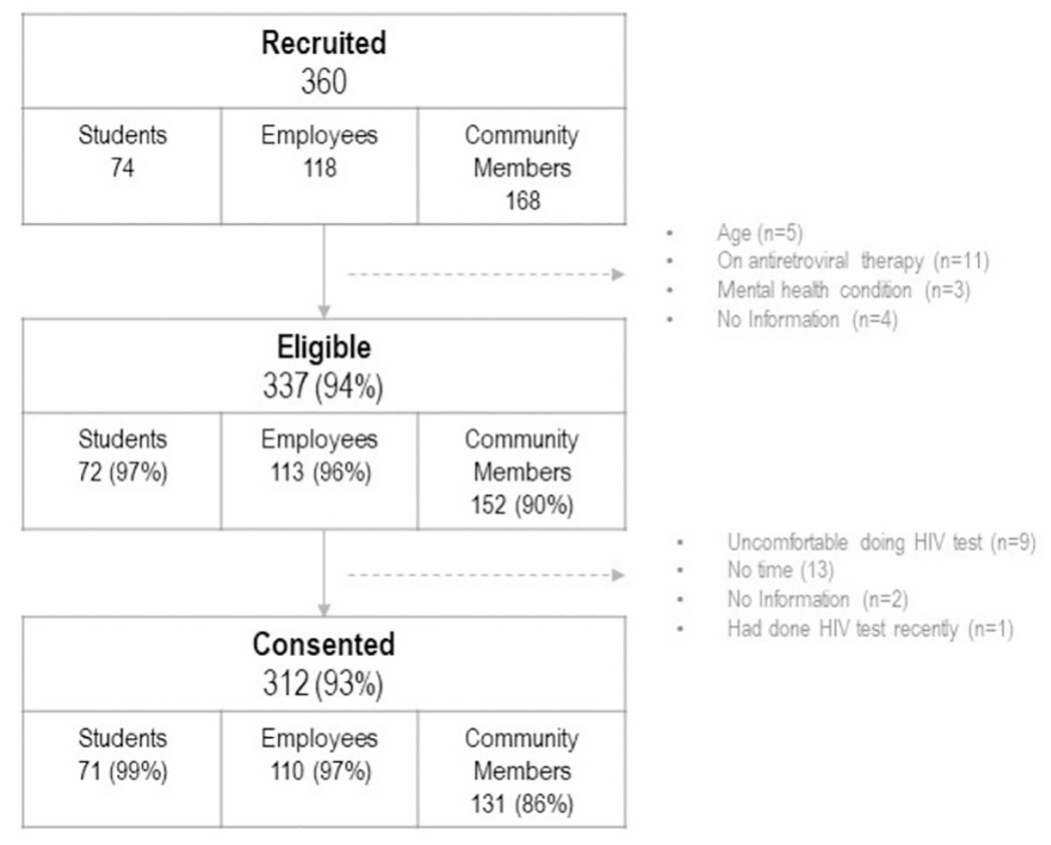
Flow chart: Number of individuals recruited, eligible and consented for study participation.

We recruited 360 individuals, with 337 (94%) being eligible to participate in study activities. Among those eligible, 312 (93%) people consented and participated in the study, broken down as follows: 110 (35%) employees, 71 (23%) students, and 131 (42%) community members. Refusal rate was 7%, mainly among community members, related to the individuals not feeling comfortable doing an HIV test as part of the study procedures, or not having enough time (Fig 1).

Most participants were male (239, 77%). Among participants, 99 (32%) reported that Portuguese was their native language; 223 (71%) stated their reading abilities in Portuguese as “good” to “very good”. The vast majority (83%) of participants reported that they had previously been tested for HIV, with approximately one fifth (19%) stating that their prior HIV testing took place more than two years prior to data collection (Table 1).

**Table 1:** Characteristics of participants of performance evaluation (n = 312).

	Employees (n = 110)	Students (n = 71)	Community Members (n = 131)	Total (n = 312)	p-value^α^
Sex (n, %)					**<0.001**
Female	13 (11.8%)	12 (16.9%)	48 (36.6%)	73 (23.4%)	
Male	97 (88.2%)	59 (83.1%)	83 (63.4%)	239 (76.6%)	
Age, years, mean (sd)	31.6 (11.6)	22.2 (2)	27.9 (9.7)	27.9 (10)	**<0.001**
Age groups, years:					**<0.001**
18–24 years	38 (34.5%)	61 (85.9%)	65 (49.6%)	164 (52.6%)	
25–34 years	39 (35.5%)	10 (14.1%)	40 (30.5%)	89 (28.5%)	
35–44 years	15 (13.6%)	0 (0%)	14 (10.7%)	29 (9.3%)	
>44 years	18 (16.4%)	0 (0%)	12 (9.2%)	30 (9.6%)	
District of residence (n, %) (1 missing)					0.998
Alto Molócuè	55 (50%)	35 (50%)	65 (49.6%)	155 (49.8%)	
Mocuba	55 (50%)	35 (50%)	66 (50.4%)	156 (50.2%)	
Educational level (n, %) (1 missing)					**<0.001**
Never went to school or had alphabetization	14 (12.7%)	0 (0%)	15 (11.5%)	29 (9.3%)	
Primary school (7^th^ grade)	24 (21.8%)	0 (0%)	34 (26.2%)	58 (18.6%)	
Basic school (10^th^ grade)	24 (21.8%)	8 (11.3%)	38 (29.2%)	70 (22.5%)	
Secondary school (12^th^ grade) / Superior	48 (43.6%)	63 (88.7%)	43 (33.1%)	154 (49.5%)	
Religion (n, %) (7 missing)					0.319
Catholic	57 (52.8%)	41 (57.7%)	61 (48.4%)	159 (52.1%)	
Muslim	10 (9.3%)	11 (15.5%)	14 (11.1%)	35 (11.5%)	
Protestant	41 (38%)	19 (26.8%)	51 (40.5%)	111 (36.4%)	
Employment status (n, %) (1 missing)					**<0.001**
Unemployed	0 (0%)	24 (33.8%)	37 (28.2%)	61 (19.6%)	
Employed / self-employed	109 (100%)	47 (66.2%)	94 (71.8%)	250 (80.4%)	
Marital status (n, %)					**<0.001**
Married/Living together	78 (70.9%)	4 (5.6%)	50 (38.2%)	132 (42.3%)	
Single (not living with partner)	30 (27.3%)	67 (94.4%)	68 (51.9%)	165 (52.9%)	
Divorced/ Widowed	2 (1.8%)	0 (0%)	13 (9.9%)	15 (4.8%)	
Native language (n, %)					**0.033**
Portuguese	30 (27.3%)	17 (23.9%)	52 (39.7%)	99 (31.7%)	
Other	80 (72.7%)	54 (76.1%)	79 (60.3%)	213 (68.3%)	
Portuguese language reading skills (n, %)					**<0.001**
Does not read / reads poorly	8 (7.3%)	5 (7%)	10 (7.6%)	23 (7.4%)	
Reads moderately	24 (21.8%)	3 (4.2%)	39 (29.8%)	66 (21.2%)	
Reads well	44 (40%)	22 (31%)	52 (39.7%)	118 (37.8%)	
Reads very well	34 (30.9%)	41 (57.7%)	30 (22.9%)	105 (33.7%)	
Ever did a HIV test (n, %)					0.16
No	14 (12.7%)	10 (14.1%)	28 (21.4%)	52 (16.7%)	
Yes	96 (87.3%)	61 (85.9%)	103 (78.6%)	260 (83.3%)	
Place of last HIV test (among those reported having tested) (n, %)					**<0.001**
HF in selected district	44 (45.8%)	29 (47.5%)	71 (68.9%)	144 (55.4%)	
HF in another district	10 (10.4%)	26 (42.6%)	23 (22.3%)	59 (22.7%)	
Other	42 (43.8%)	6 (9.8%)	9 (8.7%)	57 (21.9%)	
Timing of last HIV test (among those reported having tested (n, %)					0.053
< 3 months ago	38 (39.6%)	15 (24.6%)	20 (19.4%)	73 (28.1%)	
3–5 months ago	13 (13.5%)	11 (18%)	17 (16.5%)	41 (15.8%)	
6–11 months ago	17 (17.7%)	13 (21.3%)	23 (22.3%)	53 (20.4%)	
12–23 months ago	14 (14.6%)	14 (23%)	16 (15.5%)	44 (16.9%)	
More than 2 years ago	14 (14.6%)	8 (13.1%)	27 (26.2%)	49 (18.8%)	

Sd standard deviation; HF Health Facility

^**α**^Chi-square test (or Fisher exact test when the frequency for certain category was less than 5) for the categorical variables; ANOVA for the continuous variables.

### Observation

Among participants, 181 (58%) read the provided written instructions, of whom 22 (12%) experiencing difficulties reading the instructions, as reported by the observing research assistant. Although not included in the usability index calculation, 309 (99.4%) participants were observed to have watched the HIVST instructional video. The average UI was 80% among employees, 86% among students, and 77% among community members (Table 2). Analysis by sex and by age group showed a slightly lower UI among females (77.8%) compared to males (80.4%); and a better performance among younger people (84.6% among age group 18–24 years of age versus 62.5% among age group >44 years of age) (S1 Table). The main performance errors observed included incorrect tube positioning into the stand (152, 49%), incorrect specimen collection (134, 43%), and improper waiting time for result interpretation (130, 42%) (Table 2).

**Table 2:** Average usability index, per priority population.

	Activity: Did the participant… (expected response for UI calculation) ^α^:	Employees (n = 110) (n,%)^β^	Students (n = 71) (n,%) ^β^	Community Members (n = 131) (n,%) ^β^	Total (n = 312) (n,%) ^β^
General	read the instructions? (Y)	69 (62.7%)	44 (62.9%)	68 (51.9%)	181 (58.2%)
	show difficulties in reading instructions (of those who read instructions? (N)	58 (84.1%)	42 (95.5%)	58 (86.6%)	158 (87.8%)
	perform the test steps in correct order (steps 1–4)? (Y)	101 (91.8%)	70 (98.6%)	119 (91.5%)	290 (93.2%)
Step 1—Preparation	have difficulties in opening the components in the box? (N)	94 (85.5%)	59 (83.1%)	93 (71.5%)	246 (79.1%)
	remove the cap from the test tube? (Y)	110 (100%)	71 (100%)	127 (97.7%)	308 (99.0%)
	put the holder correctly (horizontally) on the table? (Y)	102 (92.7%)	71 (100%)	120 (92.3%)	293 (94.2%)
	**put the tube correctly in the support (45-degree angle)?** (Y)	**49 (44.5%)**	**46 (64.8%)**	**63 (48.8%)**	**158 (51.0%)**
	have any difficulties with the test tube? (N)	85 (78.0%)	61 (85.9%)	89 (68.5%)	235 (75.8%)
	remove the test device from the package? (Y)	110 (100%)	71 (100%)	130 (100%)	311 (100%)
Step 2—Collection	touch the flat pad? (N)	92 (83.6%)	69 (97.2%)	109 (83.8%)	270 (86.8%)
	**collect the sample correctly? (2 missing)** (Y)	**62 (56.4%)**	**44 (62.0%)**	**70 (54.3%)**	**176 (56.8%)**
Step 3—Mixing	place the test stick correctly in the test tube? (Y)	108 (99.1%)	71 (100%)	123 (94.6%)	302 (97.4%)
Step 4—Reading Result	**wait the correct period of time for reading (20 to 40 minutes)?** (Y)	**59 (54.1%)**	**49 (69.0%)**	**70 (54.7%)**	**178 (57.8%)**
	**Average Usability Index**	**79.4%**	**86.1%**	**76.6%**	**79.8%**

UI: Usability Index.

^**α**^:Expected response for UI calculation: *Y = Yes; N = No*.

^**β**^ Percentages of expected responses for each activity indicated the usability level for that activity. All percentages were averaged within each group to obtain the general UI for that group.

**Bold**: those with lowest usability during preparation, collection and reading of HIVST.

### Interpretation

All participants received three anonymous, predefined HIVST results using photos and were asked to provide the interpretation of the result. A total of 936 test results were presented (330, 213, and 393 results presented to employees, students, and community members, respectively), with an overall correct interpretation rate of 88% (823/936). When disaggregated by group, community members had a lower correct interpretation rate (84%; 332/393), with students performing the best (95%; 203/213) (S2 Table). When examining the results interpretation performance at the individual participant level, 75% (n = 234) of all participants responded correctly to all three presented results, with students having an overall higher result interpretation performance rate (63, 89%). Nine (3%) people failed in all three presented results (3 employees, 6 community members). Overall, 36 (12%) provided a false non-reactive result interpretation, 21 (7%) provided a false reactive result interpretation, and 14 (4%) provided both false non-reactive and false reactive result interpretations. Community members generally had a lower performance and students consistently performed best overall (Table 3).

**Table 3:** Interpretation results of pre-defined HIVST results, per priority population.

	Employees (n = 110)	Students (n = 71)	Community Members (n = 131)	Total (n = 312)	p-value^α^
**Correct interpretations for all three tests (n,%)**					0.08
0	3 (2.7%)	0 (0%)	6 (4.6%)	9 (2.9%)	
1	7 (6.4%)	2 (2.8%)	8 (6.1%)	17 (5.5%)	
2	19 (17.3%)	6 (8.5%)	27 (20.6%)	52 (16.7%)	
3	81 (73.6%)	63 (88.7%)	90 (68.7%)	234 (75%)	
**False non-reactive interpretation (n, %)** ^β^					0.08
Yes	14 (12.7%)	3 (4.2%)	19 (14.5%)	36 (11.5%)	
No	96 (87.3%)	68 (95.8%)	112 (85.5%)	276 (88.5%)	
**False reactive interpretation (n, %)** ^γ^					**0.03**
Yes	6 (5.5%)	1 (1.4%)	14 (10.7%)	21 (6.7%)	
No	104 (94.5%)	70 (98.6%)	117 (89.3%)	291 (93.3%)	
**False reactive and false non-reactive interpretation (n, %)** ^β^^,^ ^γ^					0.22
Yes	4 (3.6%)	1 (1.4%)	9 (6.9%)	14 (4.5%)	
No	106 (96.4%)	70 (98.6%)	122 (93.1%)	298 (95.5%)	

^**α**^ Fisher exact test

^β^ False non-reactive interpretation was defined as an interpretation given as non-reactive while the test result was actually reactive or invalid

^γ^ False reactive interpretation was defined as an interpretation given as reactive while the test result was actually non-reactive or invalid

## Discussion

This HIVST performance evaluation among employees, students, and community members in Mozambique showed that there were some challenges in following the correct procedures. Completing procedures incorrectly could have consequences such as invalid results and/or inaccurate interpretation. Important errors observed included incorrect sample collection procedures and not following the wait time instructions prior to result interpretation. Similar difficulties in performance using this same HIVST were seen in South-Africa: namely, removing the test tube, difficulty in placing the test tube in the stand, touching the flat pad, as well as difficulties in sample collection [5, 6]. However, despite the procedural errors, the overall usability in our study was quite favorable (80%). Other research groups have utilized different HIVST test kits, and slightly different questionnaires where the main steps were similarly observed but having subtle differences in a few of the individual steps, resulting in a usability calculation of 10 to 13 steps [5, 6, 11, 21, 22], while others only used main steps for the usability calculation [7, 8]. Overall, the usability index was high in these studies (reported scores between 85%-99%) [5, 6, 11, 21], with some studies not providing a UI score but still reporting the proportion of errors during HIVST procedures (ranging between 15–30%) [7, 8, 22]. We observed that many participants in our study did not read (or did not fully read) the instructions. The authors suspect that participants were in a hurry and wanted to spend as little time as possible on the study activities. It would be important to further understand if there are language or comprehension challenges that need to be addressed and included in communication and/or education strategies when promoting HIVST. On the other hand, continuous educational sessions and incorporation of the use of social media to regularly explain procedures might enhance knowledge. Educational sessions on HIVST procedures could be incorporated into community outreach activities and included in health promotion campaigns, for example, during community HIV counseling and testing, and index-case testing activities. Promotional videos or messages could be created which include information on HIVST procedures, including alerts on the main issues that might come up when performing the test such as the required waiting time for test results, handling of test materials (specifically, not touching the swab), and the need for test result confirmation.

A false non-reactive result interpretation was given by 12% of the participants, which (if interpreting one’s own results) could place the person at risk for not properly identifying a reactive HIVST result, and for missing the need for confirmatory testing and/or initiating necessary care and treatment. The proportion of false non-reactive result interpretation was higher among community members, potentially putting sexual partners at risk when available HIV prevention methods are not used. False reactive result interpretation was also more frequent among community members than other participant groups, though the occurrence was less frequent than for false non-reactive results. However, if effective linkage mechanisms are put in place and adhered to by HIVST users, a confirmation test can easily correct this false reactive interpretation error.

The MOH adopted a national community-based HIV self-testing strategy as of December 2021. Individuals can receive an HIVST and perform it at home or in assisted fashion with a counselor, whatever the individual prefers, but all reactive results need to be confirmed at the health facility, in accordance to existing national HIV counseling and testing guidelines [23]. Our results showed that continuous demonstration of HIVST procedures through different formats (*e*.*g*., written instructions, video demonstration, community education sessions) is necessary to maintain quality performance of test procedures. Regular performance evaluations such as the one in our study could be integrated into training and longitudinal quality assurance activities (usually organized by Ministry of Health) to ensure proper HIVST result interpretation. Social media platforms and hotlines are other viable sources of communication for such messaging, as well as information obtained through community- or health facility-based talks.

Limitations of the research included the restricted location of the study (only two rural districts in one province). Consequently, the results are not necessarily generalizable for the entire country. It is possible that the Hawthorne effect could have influenced participant behavior during the observations, resulting in overperformance. In addition, the manufacturers’ instructions are only available in Portuguese, which could have influenced HIVST performance for participants who do not speak and/or are not literate in Portuguese, despite containing clear illustrations.

## Conclusions

The study showed that usability of HIVST in rural Mozambique was favorable overall, despite some procedural errors, confirming results of other studies. Regular assessments using these validated instruments can ensure monitoring of HIVST performance quality. In addition, continuous communication and education strategies in the community and workplace seem to be necessary to ensure correct interpretation and to avoid missed opportunities for linkage to services.

## Supporting information

S1 Table Usability index, per sex and per age group. (DOCX)

S2 Table Interpretation of HIVST results, by test. (DOCX)

S1 File Questionnaire on inclusivity in global research. (DOCX)

S2 File STROBE checklist. (DOC)

S1 Data Study dataset. (DOCX)

## References

[1] WHO. Guidelines on HIV self-testing and partner notification. Supplement to consolidated guidelines on HIV testing services. December 2016 Geneva: WHO; 2016 [https://apps.who.int/iris/handle/10665/251655. 27977094

[2] WHO. WHO recommends HIV self-testing—evidence update and considerations for success Geneva: WHO; 2019 [https://www.who.int/publications/i/item/WHO-CDS-HIV-19.36.

[3] Kurth AE, Cleland CM, Chhun N, Sidle JE, Were E, Naanyu V, et al. Accuracy and Acceptability of Oral Fluid HIV Self-Testing in a General Adult Population in Kenya. AIDS Behav. 2016;20(4):870–9. doi: 10.1007/s10461-015-1213-9 26438487 PMC4799243

[4] Zanolini A, Chipungu J, Vinikoor MJ, Bosomprah S, Mafwenko M, Holmes CB, et al. HIV Self-Testing in Lusaka Province, Zambia: Acceptability, Comprehension of Testing Instructions, and Individual Preferences for Self-Test Kit Distribution in a Population-Based Sample of Adolescents and Adults. AIDS Res Hum Retroviruses. 2018;34(3):254–60. doi: 10.1089/AID.2017.0156 28969432 PMC5863088

[5] Majam M, Mazzola L, Rhagnath N, Lalla-Edward ST, Mahomed R, Venter WDF, et al. Usability assessment of seven HIV self-test devices conducted with lay-users in Johannesburg, South Africa. PLoS One. 2020;15(1):e0227198. doi: 10.1371/journal.pone.0227198 31935228 PMC6959591

[6] Deville W, Tempelman H. Feasibility and robustness of an oral HIV self-test in a rural community in South-Africa: An observational diagnostic study. PLoS One. 2019;14(4):e0215353. doi: 10.1371/journal.pone.0215353 30986228 PMC6464222

[7] Saunders J, Brima N, Orzol M, Phillips L, Milinkovic A, Carpenter G, et al. Prospective observational study to evaluate the performance of the BioSure HIV Self-Test in the hands of lay users. Sex Transm Infect. 2018;94(3):169–73. doi: 10.1136/sextrans-2017-053231 28924053

[8] Ortblad KF, Kibuuka Musoke D, Ngabirano T, Nakitende A, Taasi G, Barresi LG, et al. HIV self-test performance among female sex workers in Kampala, Uganda: a cross-sectional study. BMJ Open. 2018;8(11):e022652. doi: 10.1136/bmjopen-2018-022652 30413504 PMC6231563

[9] Pant Pai N, Balram B, Shivumar S, Martinez-Cajas J, Claessens C, Lambert G, et al. Head-to-head comparison of accuracy of a rapid point-of-care HIV test with oral versus whole-blood specimens: a systematic review and meta-analysis. Lancet Infect Dis. 2012;12:373–80. doi: 10.1016/S1473-3099(11)70368-1 22277215

[10] Belza MJ, Rosales-Statkus ME, Hoyos J, Segura P, Ferreras E, Sanchez R, et al. Supervised blood-based self-sample collection and rapid test performance: a valuable alternative to the use of saliva by HIV testing programmes with no medical or nursing staff. Sex Transm Infect. 2012;88(3):218–21. doi: 10.1136/sextrans-2011-050131 22328646

[11] Tonen-Wolyec S, Sarassoro A, Muwonga Masidi J, Twite Banza E, Nsiku Dikumbwa G, Maseke Matondo DM, et al. Field evaluation of capillary blood and oral-fluid HIV self-tests in the Democratic Republic of the Congo. PLoS One. 2020;15(10):e0239607. doi: 10.1371/journal.pone.0239607 33017442 PMC7535027

[12] INS. Mozambique—Population-based HIV Impact Assessment. INSIDA 2021 Maputo2022 [https://ins.gov.mz/divulgados-resultados-do-inquerito-sobre-o-impacto-do-hiv-e-sida-em-mocambique/. 10.1007/s10461-025-04699-7 PMC13282113 40205309

[13] INS, INE, ICF. Inquérito de Indicadores de Imunização, Malária e HIV/SIDA Em Moçambique 2015. Relatório Preliminar de Indicadores de HIV. 2015 [http://www.ins.gov.mz/images/IMASIDA/IMASIDA

[14] De Schacht C, Lucas C, Paulo P, Van Rompaey S, Fernando AN, Chinai JE, et al. Reaching Men and Young Adults in a Pharmacy-Based HIV Self-Testing Strategy: Results from an Acceptability Study in Mozambique. AIDS Res Hum Retroviruses. 2022;38(8):622–30. doi: 10.1089/AID.2021.0116 35579964 PMC11288794

[15] MOH. Guião para a Autotestagem de HIV em Moçambique Maputo2019 [https://www.misau.gov.mz/index.php/guioes-de-prevencao-e-de-cuidados-e-tratamento.

[16] INE. Population Census—Projections Population Zambézia 2017–2050 Maputo2017 [http://www.ine.gov.mz/iv-rgph-2017/projeccoes-da-populacao-2017-2050/zambezia.xls/view

[17] UNITAID. HIV rapid diagnostic tests for self-testing, 4th edition 2018 [https://unitaid.org/assets/HIVST-landscape-report.pdf.

[18] WHO. WHO Prequalification of In Vitro Diagnostics—OraQuick HIV Self-Test. Geneva2017 [http://www.who.int/diagnostics_laboratory/evaluations/pq-list/170720_final_amended_pqdx_0159_055_01_oraquick_hiv_self_test_v2.pdf.

[19] Simwinga M, Kumwenda MK, Dacombe RJ, Kayira L, Muzumara A, Johnson CC, et al. Ability to understand and correctly follow HIV self-test kit instructions for use: applying the cognitive interview technique in Malawi and Zambia. J Int AIDS Soc. 2019;22 Suppl 1:e25253. doi: 10.1002/jia2.25253 30907496 PMC6432102

[20] Team RC. R: A language and environment for statistical computing. R Foundation for Statistical Computing Vienna, Austria2022 [https://www.R-project.org/.

[21] Majam M, Rhagnath N, Msolomba V, Singh L, Urdea MS, Lalla-Edward ST. Assessment of the Sedia HIV Self-Test Device: Usability and Performance in the Hands of Untrained Users in Johannesburg, South Africa. Diagnostics (Basel). 2021;11(10). doi: 10.3390/diagnostics11101816 34679514 PMC8534357

[22] Lippman SA, Gilmore HJ, Lane T, Radebe O, Chen YH, Mlotshwa N, et al. Ability to use oral fluid and fingerstick HIV self-testing (HIVST) among South African MSM. PLoS One. 2018;13(11):e0206849. doi: 10.1371/journal.pone.0206849 30408055 PMC6224086

[23] MOH. Guião de Cuidados de HIV do Adulto, Adolescente Grávida, Lactante e Criança 2023 [https://comitetarvmisau.co.mz/docs/guiao_tarv/Tratamento%20antiretroviral%20e%20infecc%CC%A7o%CC%83es%20oportunistas%20do%20adulto,%20adolescente%20e%20crianc%CC%A7a_27.03.2023.pdf.

