# Supplementary material for: HIV self-test performance evaluation among priority populations in rural Mozambique: Results from a community-based observational study
Source: PLoS One. 2024 Jun 17;19(6):e0305391. doi: 10.1371/journal.pone.0305391 (PMC11182534; doi:10.1371/journal.pone.0305391)
Supplement: S1 Table — (DOCX) [file pone.0305391.s001.docx]

**S1 Table**. **Usability Index, per sex (a) and per age group (b).**

a.

|  | Activity**^α^**: Did the participant… (expected response for UI calculation)^α^ | Female (n=73) **^β^** | Male (n=238) **^β^** |
| --- | --- | --- | --- |
| General | read the instructions? (Y) | 37 (50.7%) | 144 (60.5%) |
|  | show difficulties in reading instructions (among those who read instructions)? (N) | 32 (88.9%) | 126 (87.5%) |
|  | perform the test steps in correct order (steps 1-4)? (Y) | 69 (94.5%) | 221 (92.9%) |
| Step 1 - Preparation | have difficulties in opening the components in the box? (N) | 52 (71.2%) | 194 (81.5%) |
|  | remove the cap from the test tube? (Y) | 71 (97.3%) | 237 (99.6%) |
|  | put the holder correctly (horizontally) on the table? (Y) | 67 (91.8%) | 226 (95.0%) |
|  | put the tube correctly in the support (45-degree angle)? (Y) *(1 missing)* | 39 (53.4%) | 119 (50.2%) |
|  | have any difficulties with the test tube? (N) *(1 missing)* | 49 (67.1%) | 186 (78.5%) |
|  | remove the test device from the package? (Y) | 73 (100%) | 238 (100%) |
| Step 2 – Collection | touch the flat pad? (N) | 62 (84.9%) | 208 (87.4%) |
|  | collect the sample correctly? (Y) *(1 missing)* | 46 (63.0%) | 130 (54.9%) |
| Step 3 - Mixing | place the test stick correctly in the test tube? (Y) *(1 missing)* | 70 (95.9%) | 232 (97.9%) |
| Step 4 – Reading (result) | wait the correct period of time for reading (20 to 40 minutes)? (Y) *(3 missing)* | 37 (52.1%) | 141 (59.5%) |
|  | **Average** | 77.8% | 80.4% |

***^α^****:Expected responses for UI calculation: Y=Yes; N=No.* ***^β^*** *Percentages reflect the Usability Index*

***Bold****: those with lowest usability index during preparation, collection and reading of HIVST (excluding the general items).*

b.

|  | Activity**^α^**: Did the participant… (expected response for UI calculation)^α^ | 18-24 yrs (n=164) **^β^** | 25-34 yrs (n=89) **^β^** | 35-44 yrs (n=29) **^β^** | >44 yrs (n=30) **^β^** |
| --- | --- | --- | --- | --- | --- |
| General | read the instructions? (Y) | 99 (60.7%) | 52 (58.4%) | 15 (51.7%) | 15 (50.0%) |
|  | show difficulties in reading instructions (among those who read the instructions)? (N) | 93 (93.9%) | 46 (88.5%) | 10 (66.7%) | 9 (64.3%) |
|  | perform the test steps in correct order (steps 1-4)? (Y) | 161 (98.2%) | 84 (94.4%) | 24 (82.8%) | 21 (72.4%) |
| Step 1 - Preparation | have difficulties in opening the components in the box? (N) | 133 (81.1%) | 74 (83.1%) | 22 (75.9%) | 17 (58.6%) |
|  | remove the cap from the test tube? (Y) | 164 (100%) | 87 (97.8%) | 28 (96.6%) | 29 (100%) |
|  | put the holder correctly (horizontally) on the table? (Y) | 162 (98.8%) | 86 (96.6%) | 23 (79.3%) | 22 (75.9%) |
|  | put the tube correctly in the support (45-degree angle)? (Y) | 98 (59.8%) | 42 (47.2%) | 11 (39.3%) | 7 (24.1%) |
|  | have any difficulties with the test tube? (N) | 137 (83.5%) | 70 (78.7%) | 16 (55.2%) | 12 (42.9%) |
|  | remove the test device from the package? (Y) | 164 (100%) | 89 (100%) | 29 (100%) | 29 (100%) |
| Step 2 – Collection | touch the flat pad? (N) | 152 (92.7%) | 80 (89.9%) | 22 (75.9%) | 16 (55.2%) |
|  | collect the sample correctly? (2 missing) (Y) | 105 (64.4%) | 46 (51.7%) | 14 (48.3%) | 11 (37.9%) |
| Step 3 - Mixing | place the test stick correctly in the test tube? (Y) | 164 (100%) | 86 (96.6%) | 26 (89.7%) | 26 (92.9%) |
| Step 4 – Reading (result) | wait the correct period of time for reading (20 to 40 minutes)? (Y) | 107 (66.5%) | 47 (52.8%) | 13 (44.8%) | 11 (37.9%) |
|  | **Average** | 84.6% | 79.7% | 69.7% | 62.5% |

***^α^****:Expected responses for UI calculation: Y=Yes; N=No.* ***^β^*** *Percentages reflect the Usability Index*

***Bold****: those with lowest usability index during preparation, collection and reading of HIVST (excluding the general items).*
