# Supplementary material for: HIV self-test performance evaluation among priority populations in rural Mozambique: Results from a community-based observational study
Source: PLoS One. 2024 Jun 17;19(6):e0305391. doi: 10.1371/journal.pone.0305391 (PMC11182534; doi:10.1371/journal.pone.0305391)
Supplement: S2 Table — (DOCX) [file pone.0305391.s002.docx]

**S2 Table**. **Interpretation per test, by study group.**

|  | **Employees** | **Students** | **Community** | **[ALL]** | **p-value*** |
| --- | --- | --- | --- | --- | --- |
| **By test(s):** |  |  |  |  |  |
|  | **N=330** | **N=213** | **N=393** | **N=996** |  |
| **Test 1:** |  |  |  |  | 0.134 |
| Correct | 90 (81.8%) | 67 (94.4%) | 110 (84%) | 267 (85.6%) |  |
| Wrong | 16 (14.5%) | 3 (4.2%) | 18 (13.7%) | 37 (11.9%) |  |
| Don't Know Result | 4 (3.6%) | 1 (1.4%) | 3 (2.3%) | 8 (2.6%) |  |
| **Test 2:** |  |  |  |  | **0.024** |
| Correct | 100 (90.9%) | 69 (97.2%) | 108 (82.4%) | 277 (88.8%) |  |
| Wrong | 7 (6.4%) | 2 (2.8%) | 16 (12.2%) | 25 (8%) |  |
| Don't Know Result | 3 (2.7%) | 0 (0%) | 7 (5.3%) | 10 (3.2%) |  |
| **Test 3:** |  |  |  |  | 0.244 |
| Correct | 98 (89.1%) | 67 (94.4%) | 114 (87%) | 279 (89.4%) |  |
| Wrong | 7 (6.4%) | 4 (5.6%) | 14 (10.7%) | 25 (8%) |  |
| Don't Know Result | 5 (4.6%) | 0 (0%) | 3 (2.3%) | 8 (2.6%) |  |
| **All three tests** |  |  |  |  |  |
| Correct | 288 (87%) | 203 (95%) | 332 (84%) | 823 (88%) | **<0.001** |
| Wrong | 30 (9%) | 9 (4%) | 48 (12%) | 87 (9%) |  |
| Don’t know result | 12 (4%) | 1 (0%) | 13 (3%) | 26 (3%) |  |

*Fisher exact test
