# Supplementary material for: HIV self-test performance evaluation among priority populations in rural Mozambique: Results from a community-based observational study
Source: PLoS One. 2024 Jun 17;19(6):e0305391. doi: 10.1371/journal.pone.0305391 (PMC11182534; doi:10.1371/journal.pone.0305391)
Supplement: S1 Data — (DOCX) [file pone.0305391.s005.docx]

**S1 Data – Link to dataset**

Link: <https://osf.io/czd7n/?view_only=ed6999175e134649b36abf0eaa695b0e>
